# Dysfunctional counting of mental time in Parkinson’s disease

**DOI:** 10.1038/srep25421

**Published:** 2016-05-05

**Authors:** Motoyasu Honma, Takeshi Kuroda, Akinori Futamura, Azusa Shiromaru, Mitsuru Kawamura

**Affiliations:** 1 Department of Neurology, Showa University School of Medicine, 1-5-8 Hatanodai, Shinagawa-ku, Tokyo 142-8666, Japan

## Abstract

Patients with Parkinson’s disease (PD) often underestimate time intervals, however it remains unclear why they underestimate rather than overestimate them. The current study examined time underestimation and counting in patients with PD, in relation to dopamine transporter (DaT) located on presynaptic nerve endings in the striatum. Nineteen non-dementia patients with PD and 20 age- and sex-matched healthy controls performed two time estimation tasks to produce or reproduce time intervals with counting in the head, to examine dysfunctional time counting processing. They also performed tapping tasks to measure cycles of counting with 1 s interval with time estimation. Compared to controls, patients underestimated time intervals above 10 s on time production not reproduction tasks, and the underestimation correlated with fast counting on the tapping task. Furthermore, striatal DaT protein levels strongly correlated with underestimation of time intervals. These findings suggest that distortion of time intervals is guided by cumulative output of fast cycle counting and that this is linked with striatal DaT protein deficit.

The ability to accurately recognize and express physical time intervals is fundamentally important to everyday life. Unlike light or sound, time has no corresponding sensory organ, which suggests that it is perceived, processed, and produced by various neural networks in the brain. Previous research has found that the dorsal striatum, which consists of the putamen and caudate nucleus, plays a central role in time and rhythm perception^1^,^2^. It is known that some patients with neurological disease have distorted estimation of mental time, and this is particularly true for patients with Parkinson’s disease (PD)^3^,^4^. Since PD affects the striatum, it is significant that patients presenting movement disorder also underestimate time intervals.

Time processing on the second (s) time scale (rather than hours or circadian timing) is linked with at least three systems. The Scalar Expectancy Theory proposes that time is sequentially processed in three stages: clock, memory, and decision^5^,^6^, suggesting dissociable neural systems for time estimation. Underestimation of time intervals on production tasks that requires expression of specified time interval (Fig. 1a), has robust findings in patients with PD^3^,^7^,^8^,^9^, while findings from time reproduction tasks, that requires repetition of a previously presented physical time duration (Fig. 1b), have been inconsistent^7^,^10^,^11^,^12^. In view of the fact that patients with PD frequently exhibit cognitive or memory dysfunctions^13^, these conflicting findings may be due to inadequate memory control in participants. Furthermore, a damaged hippocampus results in underestimation of interval reproduction above 20 s^14^, suggesting that memory dysfunction elongates or contracts estimation of time due to forgetting the estimated starting point.

Patients with PD have significantly decreased dopamine^15^,^16^,^17^, and this is related to difficulty in time estimation^3^. Furthermore, previous studies in PD patients report that administration of a dopamine agonist leads to a shift in time interval estimation towards normal. That is, time estimation is extended by dopamine^7^,^9^. Moreover, most patients with PD have a striatal disorder involving proteins such as presynaptic dopamine transporter (DaT), responsible for incorporation and transmission of dopamine components^18^. However, it remains unclear whether there is a relationship between distorted time estimation and striatal DaT level.

Another problem with investigating interval counting is that it provides no insight into what is happening during estimation. We hypothesized that underestimation of time intervals in PD results from abnormal time counting of brief units during time estimation. This is supported by findings that patients with PD develop executive dysfunction, as revealed by disordered tapping^19^, motor planning^20^, and divided attention^21^. Cycles of subjective 1 s intervals in patients with PD may be shorter than physical 1 s intervals and the overall effect gross underestimation of the passage of physical time.

The current study had three goals. First, to examine dysfunctional time estimation separate from working memory ability, we compared time production and reproduction tasks after screenings for cognitive/memory ability. Second, to examine a possible relationship between time estimation accuracy and striatal DaT protein levels, we used DaT imaging on presynaptic nerve endings in striatum^22^. Third, to better understand whether time underestimation of interval in patients with PD is based on fast time counting or not, we examined tapping speed during time estimation using a novel tapping task to measure cycles of 1 s counting (Fig. 1c,d). Unlike previous studies preventing participants from time counting in their heads, we focused on abnormal counting cycles in time processing in PD patients. We hypothesized that striatal DaT deficit is associated with fast counting during time estimation, and that distorted underestimation of time intervals is guided by cumulative output of fast cycle time counting independent of working memory ability.

## Results

### Striatal DaT deficit in Parkinson’s disease

In a patient without PD, DaT imaging indicated that binding radiations to DaT accumulated in the striatum in the shape of twin commas (Fig. 2a). In contrast, there was low striatal accumulation of radiation in the 19 patients with PD (Fig. 2b). One patient with scan without evidence of dopaminergic deficit (SWEDD)^23^ was observed and excluded from the data. We analyzed the striatal radiation counts (RCs) and the specific binding ratio (SBR)^24^. The RCs were not correlated with the Unified Parkinson’s Disease Rating Scale (UPDRS)^25^ (Pearson’s *r* = 0.382, *p* = 0.097). In contrast, SBR negatively correlated with the UPDRS (*r* = −0.525, *p* = 0.021) (Fig. 2c).

### Distorted estimation on time production not reproduction in Parkinson’s disease

In the time production task (Fig. 3a), there was a significant main effect of trial duration (*F*_(10,370)_ = 25.488, *p* < 0.0001, *η*^2^ = 0.408), no effect of disease (*F*_(1,37)_ = 0.647, *p* = 0.426, *η*^2^ = 0.017), and a significant interaction between these two factors (*F*_(10,370)_ = 8.487, *p* < 0.0001, *η*^2^ = 0.187) in two-way ANOVA. A post-hoc test revealed that the estimated error rates in 10 s, 20 s, 30 s, 60 s, 120 s, and 300 s trials were lower in patients with PD compared to normal controls (all *p* < 0.05). Furthermore, error rates in 0.5 s duration trials were higher in patients with PD compared to normal controls (*p* < 0.05, respectively). In the time reproduction task (Fig. 3b), there was a significant main effect of trial duration (*F*_(10,370)_ = 23.297, *p* < 0.0001, *η*^2^ = 0.386), no effect of the disease (*F*_(1,37)_ = 3.330, *p* = 0.076, *η*^2^ = 0.093), and a significant interaction (*F*_(10,370)_ = 2.740, *p* = 0.003, *η*^2^ = 0.069). A post-hoc test showed that the error rates above 1 s trials in patients with PD were the same as normal controls, whereas error rates on 0.5 s durations trials were higher than in normal controls (*p* < 0.05, respectively). Moreover, UPDRS (-total, part 2, and part 3) and Hoehn-Yahr stage negatively correlated with error rates in 10 s, 20 s, 30 s, 60 s, 120 s, and 300 s trials on the time production task, while there were no such correlations on the time reproduction task (Supplementary Tables 1 and 2).

### Fast counting on tapping action in Parkinson’s disease

Typically, tap intervals in PD patients were shorter than those in normal controls on both the tapping task with time production (Fig. 4a and Supplementary Video 1) and the simple tapping task (Fig. 4b). That is to say, tapping speed in PD patients was fast. For intervals between taps (Fig. 4c), there were significant main effects of tapping task type (*F*_(1,37)_ = 3.621, *p* = 0.049, *η*^2^ = 0.108) and presence of the disease (*F*_(1,37)_ = 17.088, *p* < 0.0001, *η*^2^ = 0.316), and there was no interaction (*F*_(1,37)_ = 1.474, *p* = 0.232, *η*^2^ = 0.038) in two-way ANOVA. Post-hoc test revealed that mean time of interval in PD was shorter than normal control on both tapping tasks (*p* < 0.0001, respectively). While the interval was longer on the tapping task with time production compared to the simple tapping task in normal control (*p* = 0.023), no such difference was found in PD (*p* = 0.859). Furthermore, for standard deviation (SD) among intervals (Fig. 4d), there was a significant main effect of disease (*F*_(1,37)_ = 4.261, *p* = 0.046, *η*^2^ = 0.103), no main effect of tapping task type (*F*_(1,37)_ = 3.607, *p* = 0.065, *η*^2^ = 0.089), and a significant interaction between these two factors (*F*_(1,37)_ = 4.885, *p* = 0.033, *η*^2^ = 0.117). Post-hoc test showed that SD on tapping with time production task was smaller for PD compared to normal control (*p* = 0.011), while there was no such difference on the simple tapping task (*p* > 0.5, respectively). The SD of tapping task with time production was greater than that of simple tapping in normal control (*p* = 0.005), but this was not the case in PD (*p* = 0.854).

### No effect of tapping action on time production

For intra-individual differences, estimated durations on tapping task with time production were no different to those on time production task of 60 s in both normal control (*t*_(18)_ = 0.909, *p* = 0.573, *η*^2^ = 0.126, Supplementary Fig. 1a) and PD (*t*_(18)_ = 0.182, *p* = 0.858, *η*^2^ = 0.086, Supplementary Fig. 1c) in paired *t* tests. Moreover, interval durations in the tapping task with time production positively correlated with the time production task of 60 s in both normal control (*r* = 0.718, *p* < 0.0001, Supplementary Fig. 1b) and PD (*r* = 0.751, *p* < 0.0001, Supplementary Fig. 1d). Within individuals, estimated durations between the two tasks were nearly identical.

### Correlation of striatal DaT deficit with underestimation on time production

The SBR in patients with PD positively correlated with error rates of durations in 10 s (*r* = 0.499, *p* = 0.030, Fig. 5a), 20 s (*r* = 0.534, *p* = 0.018, Fig. 5b), 30 s (*r* = 0.519, *p* = 0.023, Fig. 5c), 60 s (*r* = 0.561, *p* = 0.012, Fig. 5d), 120 s (*r* = 0.503, *p* = 0.028, Fig. 5e), and 300 s (*r* = 0.549, *p* = 0.015, Fig. 5f) trials in time production task, while no correlation was found in 0.5 s to 5 s (all *p* > 0.05, Supplementary Table 1). No such correlations were found in the time reproduction task (all *p* > 0.05, Supplementary Table 1).

### Delayed initial response in Parkinson’s disease

For initial response times on tapping tasks, there was a significant main effect of disease (*F*_(1,37)_ = 12.018, *p* < 0.0001, *η*^2^ = 0.312), no main effect of tapping task (*F*_(1,37)_ = 0.818, *p* = 0.598, *η*^2^ = 0.028), and no interaction between these factors (*F*_(1,37)_ = 1.056, *p* = 0.277, *η*^2^ = 0.039) in two-way ANOVA (Supplementary Fig. 2a). Multiple comparison test revealed that mean initial response time in PD was longer than that for normal control in both tapping tasks (*p* < 0.0001, respectively). The initial response negatively correlated to SBR in tapping with time production (*r* = −0.452, *p* = 0.045) (Supplementary Fig. 2b) and simple tapping tasks (*r* = −0.621, *p* = 0.003) (Supplementary Fig. 2c).

## Discussion

The current results agree with findings that patients with PD have striatal DaT reduction^22^,^26^,^27^, and reveal a time underestimation for intervals greater than 10 s in time production but not reproduction tasks. Furthermore, we found that striatal DaT deficit significantly correlated to underestimation of time intervals with counting task. Taking these results together, we suggest that underestimation of time intervals in PD is linked to dysfunctional counting of mental time, and may be associated with striatal DaT protein.

Underestimation of intervals on time production task was similar to previous results^7^,^8^,^9^, and greater underestimation was associated with greater PD severity. In contrast, there was no difference between PD and normal control on the time reproduction task. This time reproduction task is thought to depend on working memory. For example, in the time reproduction task, even when a patient estimated 60 s of sample figure as 40 s, if the patient retained and reproduced the maintained 40 s, the answer was rated as accurate. Although previous researches have reported that working memory or episodic memory in PD patients is disordered^28^, our preliminary screening selection for cognitive/memory ability may be successful and lead to accurate estimation on the time reproduction task in patients with PD. Altogether, the current results of underestimation on time production may reflect a true dysfunctional time processing, independent of working memory ability.

On a passive tapping task corresponding to presenting stimuli, patients with PD have been found to produce delayed timing and demonstrate increase in timing variability due to impaired response function^19^,^29^,^30^,^31^. However, we found that patients were able to continuously tap with a steady rhythm on our spontaneous tapping tasks. The result is similar to finding that patients with cerebellar lesions show disrupted timing of discontinuous but not continuous movements^32^. We suggest that motor function on spontaneous and continuous movements in PD patients is normal. We also found that cycles of subjective 1 s counting on simple tapping task in PD were faster than normal control. Thus, we believe that underestimation of time intervals in PD is caused by cumulative output of fast cycles in time counting. Surprisingly, tap interval variability in normal control was greater than that in PD on the tapping task with time production. Recent research indicated dysfunction of motor planning and divided attention in patients with PD^20^,^21^,^33^,^34^. For example, patients often display errors in the latter half of a trial in a pole avoidance task^20^ and choice reaction time is high on a divided attentional task^21^. In our tapping task with time production, in which participants estimated a 60 s period with subjective 1 s interval taps, both planning and divided attention were required to adjust whole time duration and to allocate attention to tapping and whole time duration. It is possible that normal controls calculated whole time duration and may have balanced tapping speed in the middle of the task, resulting in larger variances between taps. In contrast, tapping variation in patients with PD was small compared to normal controls on the tapping task with time production. The property of fixed tapping might provide a potential mechanism such as dysfunctional time planning in PD.

Earlier studies reported that underestimation of time intervals in patients with PD shifts towards normal following administration of a dopamine agonist, suggesting that it plays a role in time processing. Related to this, we found that striatal DaT, a protein that incorporates dopamine, is linked with time processing. Hitherto it has been difficult to establish a quantitative relationship between dopamine and behaviour due to differences in pharma-efficacy and time to onset. Quantification of DaT protein may thus serve as a guide to both disease severity and behaviours including time production. The fact that we found no correlation between SBR and estimation on time reproduction also suggests that striatal DaT deficit in PD is independent of working memory ability. With regard to the Scalar expectancy theory, consisting of clock (pacemaker, mode switch, and accumulator systems), memory (working memory and reference memory systems), and decision (comparator system) stages^5^,^6^, our evidence suggests that the pacemaker system is deficient in PD, while other systems and stages remains normal. Even if a patient counted errant 1 s cycles because of an impaired pacemaker system, the same patient should still be able to retain and reproduce the maintained cycles because of unimpaired working memory system. Consequently, the patient’s comparator system will judge distorted total duration as a true. Correlation between DaT with underestimation on time production task may be due to errant counting cycles linked to abnormal pacemaker system. In contrast, no correlation between DaT with time reproduction task may be due to a retention capability depending on normal working memory even when the pacemaker system is abnormal. Furthermore, counting cycle on reference memory in patients with PD may be abnormal because they have acquired new errant counting cycle though exposure to disease. Despite errant counting cycle on pacemaker and reference memory systems, unimpaired working memory system may lead to normal performance on time reproduction task.

The current study demonstrates a behavioral mechanism underlying underestimation of time intervals on counting task in patients with PD. Here we draw attention to a possible underlying neural mechanism related to striatal DaT deficit, and that time processing is produced by a number of neural networks^1^,^2^. Tremor, a characteristic symptom of PD, may provide us with a clue. Poverty of dopamine linked to deficient DaT protein in the striatum leads to dopaminergic decline in various regions^35^. Loss of striatal dopamine originated in substantia nigra leads to increase in globus pallidus output and activity cycles^36^. The subthalamic nuclei subsequently synchronize with fast cycles of the globus pallidus^37^,^38^, and these signals are sent to primary- and pre-motor cortices^39^. It is thought that, these signals on such a motor loop then cause development of tremor^37^,^40^. A cognitive loop system is similar to motor loop in that signals go through the specific route such a striatum–pallidus–thalamic–cortex^41^. When such signals originated in substantia nigra are sent to the cognitive loop^35^, prefrontal cortex processing may be influenced by cycles of the activity signals. As the result, cycles of time counting might quicken. Here we believe that abnormal counting for time estimation might be associated to higher-level function based on striatal DaT deficit.

Our study has several limitations. First, counting in the head is attention demanding and it means that the current time estimation tasks were dual tasks. Attention should allocate both counting and estimation of time interval, and temporal processing during the current task may be different from that during tasks without a counting requirement. However, underestimation on time production with/without tapping in the current study was comparable to underestimation in the previous studies^3^,^7^,^8^,^9^. This suggests that the additional attention needed for counting has no effect on estimation of time interval. A further study is required to understand detailed temporal processing, including attentional weighting to counting and time estimation. Second, both PD patients and normal controls had high error rates on 0.5 s duration trials, in both estimation tasks. It is a possible that the error rates were influenced by the task of 1 s counting. The 0.5 s duration trial should make it difficult to accurately respond because participants were asked to count in 1 s cycle. Furthermore, higher rates in PD patients, compared to normal controls, may be due to bradykinesia, resulting in delayed initial movement^42^,^43^. This view is supported by delay of initial response time in our tapping tasks, as well as data from previous research^19^,^29^. Theoretically, delayed initial response due to bradykinesia may affect all trials on estimation tasks, although this may alleviate as task duration lengthens and be stronger in the 0.5 s duration task. Therefore, high error rate on 0.5 s duration trials may be influenced by task difficulty and motor disability of PD rather than time misestimation. Finally, our study used DaT scanning as a marker of dopamine dysregulation rather raclopride scanning due to limitations in our own hospital facilities. Further research is required using raclopride scanning because of its high resolution^44^,^45^. This, we believe, will measure more accurately dopamine release during the time estimation tasks.

## Methods

### Participants

Clinical neurologists recruited 39 PD patients who met the diagnostic criteria of Parkinson’s Disease Society Brain Bank^46^, and 20 patients with no signs of dementia selected by Mini-Mental Status Examination (MMSE; score > 25)^47^,^48^ and Montreal Cognitive Assessment (MoCA; score > 25)^49^,^50^ (Table 1). A patient with SWEDD was excluded. Twenty age-matched normal controls with no current neurological disease history and no signs of dementia took part. The participants showed no abnormalities, as revealed by magnetic resonance imaging with fluid attenuated inversion recovery and diffusion-weighted imaging. PD severity was measured using UPDRS^25^, the Hoehn-Yahr scale, and disease duration. The UPDRS consists of 4 parts: mentation, behavior, and mood (part 1), activities of daily life (part 2), motor examination (part 3), and complications of therapy (part 4). We calculated each and the sum of parts 1 to 4 (total). All patients with PD had taken a dopamine agonist (L-DOPA), which had no influence on DaT imaging^26^, and they participated in behaviour experiments in the *On* condition. The study was approved by the Ethics Committee of Showa University Hospital and conducted according to the principles of the Declaration of Helsinki. All participants provided written informed consent including permission to film for publication.

### DaT imaging

DaT scanning utilizes ioflupane (^123^I-FP-CIT), a radio-iodinated cocaine analogue^26^,^27^. It has high affinity for DaT protein located on presynaptic nerve endings in the striatum. These are projections of dopaminergic neurons from the substantia nigra. Binding radiation of DaT thus reflects number of striatal dopaminergic neurons. Three hours after injection of ioflupane (167 MBq), single photon emission computed tomography imaging was performed with a triple-headed gamma camera (GCA-9300R, Toshiba Medical Systems Corporation, Tokyo, Japan), using fan beam collimators (N2). Ninety projection images were obtained over 360 degrees by rotating each head 120 degrees, following a circular contour, where the radius of rotation was minimized for each subject. The matrix size was 128 × 128, and a magnification factor of 1.00 rendered a pixel size of 1.72 mm. Radiation counts were acquired within a 10% symmetrical energy window centered around 159 keV. Image post-processing was performed using DaTView software (Nihon Medi-Physics, Tokyo, Japan). Raw projections were filtered prior to reconstruction with a Butterworth filter, cut-off 0.76 cycles/cm and order 4. Trans-axial slices covering the whole brain were reconstructed using OS-EM (4 iterations and 8 subsets), and the range covered whole brain in slice thickness of 1 pixel/slice. Each striatal volume was set up 11.2 ml in right and left. Binding radiation accumulation to DaT was expressed by radiation counts (RCs) and specific binding ratio (SBR), that is a ratio of striatum to whole brain calculated using the Bolt method^24^. The imaging was conducted within a month before/after behavioral measurement.

### Behavioral measurements

**Figure 1:**
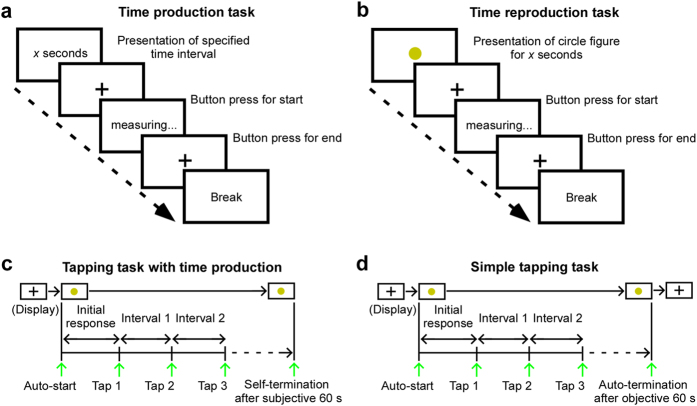
Behavior measurement. (**a**) In the time production task, a target interval time was presented at the beginning of each trial, after the number presentation disappeared from the screen, participants estimated the indicated time interval by pressing a button for start and end. (**b**) In the time reproduction task, a circle figure was presented on-screen for a given duration at the beginning of each trial, after the circle figure disappeared from the screen, participants reproduced the duration of the circle figure by pressing a button for start and end. (**c**) In the tapping task with time production, following the on-screen presentation of a circle, participants continuously tapped a button at subjective 1 s intervals and stopped after passage of a subjective period of 60 s. (**d**) In the simple tapping task, after the on-screen presentation of a circle, participants were asked to continuously tap a button at subjective 1 s intervals until automatic termination of 60 s figure presentation.

**Figure 2:**
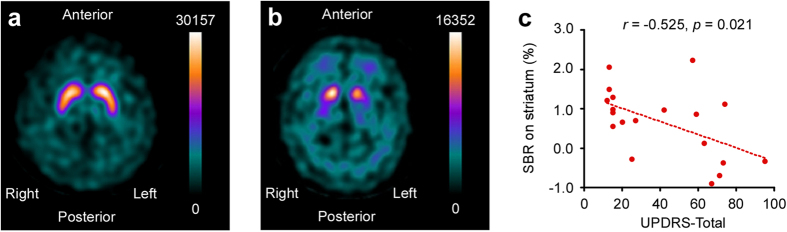
Striatal DaT deficit in PD. Binding radiation accumulation in (**a**) a sample patient with essential tremor and (**b**) patient with Parkinson’s disease (PD) (Hoehn-Yahr stage 2) in striatal DaT imaging with coronal view. The numbers indicate binding radiation counts (RCs) on striatum per pixel. (**c**) The specific binding ratio (SBR) on striatum in PD was correlated to UPDRS.

**Figure 3:**
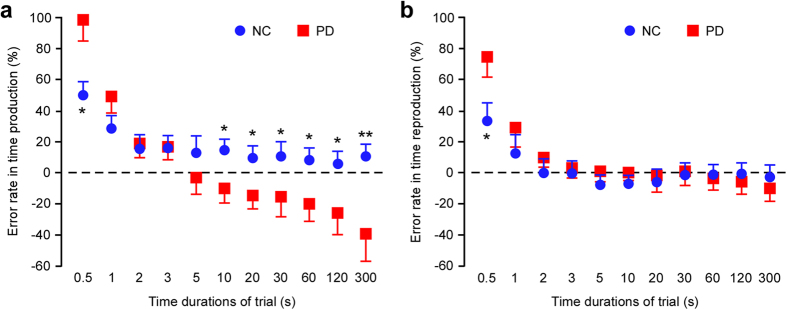
Distorted estimation in PD on time production. (**a**) Error rates above 10 s in Parkinson’s disease (PD) were lower than normal control (NC) and the error rates on 0.5 s duration intervals in PD were higher than normal control on time production task. (**b**) Time reproduction of durations greater than 2 s in PD were no different to normal controls, whereas error rates on short duration intervals were higher than normal control. A plus sign (‘+’) or minus sign (‘−’) was used to denote overestimation or underestimation of target interval. Asterisks indicate significance (**p < 0.0001, *p < 0.05). Error bars indicate the SEM.

**Figure 4:**
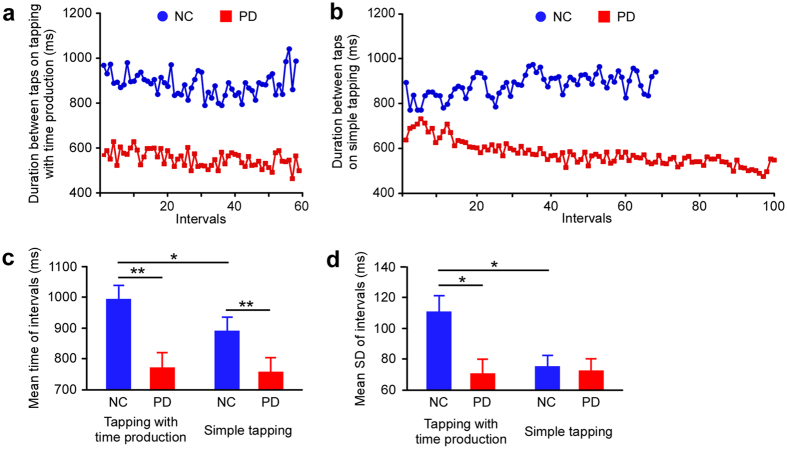
Fast and invariant counting on tapping tasks in patients with PD. (**a**) In the tapping task with time production, tapping cycles of patients with Parkinson’s disease (PD) was short and stable compared to normal controls (NC). (**b**) In the simple tapping task, tap cycles in PD were also short and stable compared to normal controls, and the short cycle led to a greater number of taps. (**c**) Mean interval on the tapping task with time production was closer to 1 s on average compared to the simple tapping task in normal control, while the intervals produced by patients with PD were faster than normal controls on both tapping tasks. (**d**) Standard deviation (SD) among intervals became large on tapping task with time production compared to simple tapping in normal controls, while there was no such difference between tapping tasks in patients with PD. Asterisks indicate significance (**p < 0.0001, *p < 0.05). Error bars indicate the SEM.

**Figure 5:**
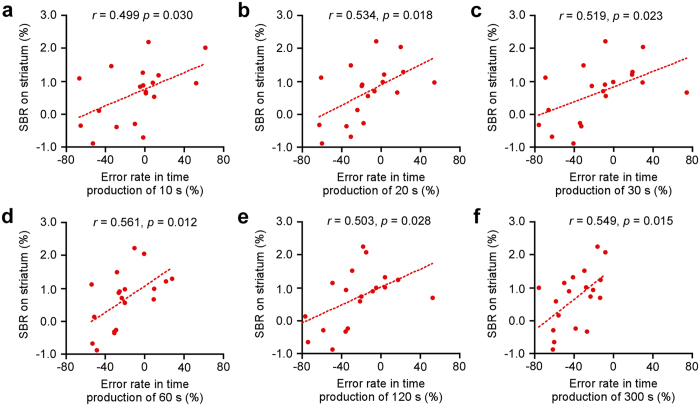
Correlation of striatal DaT deficit with underestimation on time production. A plus sign (‘+’) or minus sign (‘−’) on the horizontal axis was used to denote overestimation or underestimation of target interval. Pearson’s correlation coefficients showed the SBR on striatum in PD patients positively correlated with error rate in (**a**) 10 s, (**b**) 20 s, (**c**) 30 s, (**d**) 60 s, (**e**) 120 s, and (**f**) 300 s trials in time production task.

We used two tasks to measure estimation of time intervals. In the time production task, the duration of the interval to be produced was presented for 3 s at the beginning of each trial, after the number presentation disappeared from the screen, participants estimated the indicated time interval by pressing a button for start and end (Fig. 1a). In the time reproduction task, a circle was presented on-screen for a specific duration at the beginning of each trial, after the sample disappeared from the screen, participants reproduced the circle presentation duration by pressing a button for start and end (Fig. 1b). Participants were asked to count every 1 s in their heads. Both the production and reproduction task consisted of 11 trials of different target durations (0.5, 1, 2, 3, 5, 10, 20, 30, 60, 120, and 300 s intervals), as determined by reference to a pioneer neuropsychological study^14^. Prior to experiment, participants completed 3 practice trials of 4 s intervals, which was not used in either task. Trial order was counterbalanced among the 11 interval durations. Participants received no feedback.

There were also two kinds of tapping task. In the task with time production, after the circle had been presented on-screen, participants were asked to continuously tap a button every subjective 1 s for a subjective total duration of 60 s from figure presentation start to final tap (Fig. 1c). In the simple tapping task, participants were asked to continuously tap a button every 1 s on seeing the on-screen circle, until automatic termination of the 60 s figure presentation (Fig. 1d). There was no prior notice about trial duration on the simple tapping task. Participants received no feedback.

All behavioral experiments were conducted between 9:00 and 12:00 A.M. to eliminate any confounding effects due to circadian fluctuation in time perception^51^. For time production and time reproduction tasks, we converted time interval data into percentages based on the 11 target interval durations. A plus sign (‘+’) was used to denote an overestimation of the target interval and a minus sign (‘−’) to denote underestimation. This allowed us to compare performance across the different task durations. Four behavioral tasks (time production, reproduction, tapping with time production, and simple tapping) were counterbalanced in a session. The session was repeated three times on another days within 3 months to reduce the burden on patients because it took an hour to conduct one session. We also controlled the time for dose and experiment. The 3 trial data was averaged as a sample.

### Statistical analysis

Two-way ANOVA and multiple comparisons were conducted for performance in time estimation tasks, tapping intervals and variance, and delayed initial responses. All results are presented as means and standard errors of the mean (SEM), and effect size (*η*^2^). A *p* value of 0.05 was considered statistically significant, but a corresponding Bonferroni-adjusted *p* value for each statistical level was set for each post-hoc test. Pearson’s correlation coefficients were calculated to examine the relationships between PD symptom severity (UPDRS, Hoehn-Yahr stage, and disease duration), behavioral performance (time production and reproduction tasks), and DaT scan (RCs and SBR). All tests were two-tailed. SPSS 22.0 for Windows (IBM, Inc., Chicago, IL) was used for the statistical analyses.

## Additional Information

**How to cite this article**: Honma, M. *et al*. Dysfunctional counting of mental time in Parkinson’s disease. *Sci. Rep*. **6**, 25421; doi: 10.1038/srep25421 (2016).

## Supplementary Material

Supplementary Information

Supplementary Movie S1

## Figures and Tables

**Table 1:** Participant details.

	NC (n = 20)	PD (n = 19)	*p* value
Age (years)	68.5 (6.73)	72.0 (6.34)	0.15
Sex			
Female	11	11	–
Male	9	8	–
Hand dominance			
Right	20	19	–
Left	0	0	–
MMSE	29.1 (0.91)	28.7 (1.39)	0.223
MoCA	27.4 (1.39)	27.2 (1.23)	0.852
UPDRS			
Total	–	40.6 (27.56)	–
Part 1	–	1.6 (1.12)	–
Part 2	–	10.6 (8.88)	–
Part 3	–	24.8 (16.39)	–
Part 4	–	3.5 (3.17)	–
Hoehn-Yahr stage	–	2.7 (0.91)	–
PD duration (years)	–	7.4 (4.66)	–

MMSE: Mini-Mental State Examination (max: 30). MoCA: Montreal Cognitive Assessment (max: 30). UPDRS: Unified Parkinson’s Disease Rating Scale. Total: The sum of Part 1 to 4. Part 1: Mentation, behavior, and mood. Part 2: Activities of daily life. Part 3: Motor Examination. Part 4: Complications of therapy. The standard deviations are shown in parentheses. Pearson’s *X*^2^ test revealed no significant difference in the ratio of sex between groups (*X*^2^ = 0.001, *P* = 0.904). Unpaired *t* tests were used for between-group comparisons. NC = normal control. PD = Parkinson’s disease.
