# Supplementary material for: Dysfunctional counting of mental time in Parkinson’s disease
Source: Sci Rep. 2016 May 5;6:25421. doi: 10.1038/srep25421 (PMC4857080; doi:10.1038/srep25421)
Supplement: Supplementary Information [file srep25421-s1.pdf]

Supplementary Information

Dysfunctional counting of mental time in Parkinson's disease

Motoyasu Honma, Takeshi Kuroda, Akinori Futamura, Azusa Shiromaru, Mitsuru Kawamura

Supplementary Figures 1–2.

Supplementary Tables 1–2.

Supplementary Video 1.

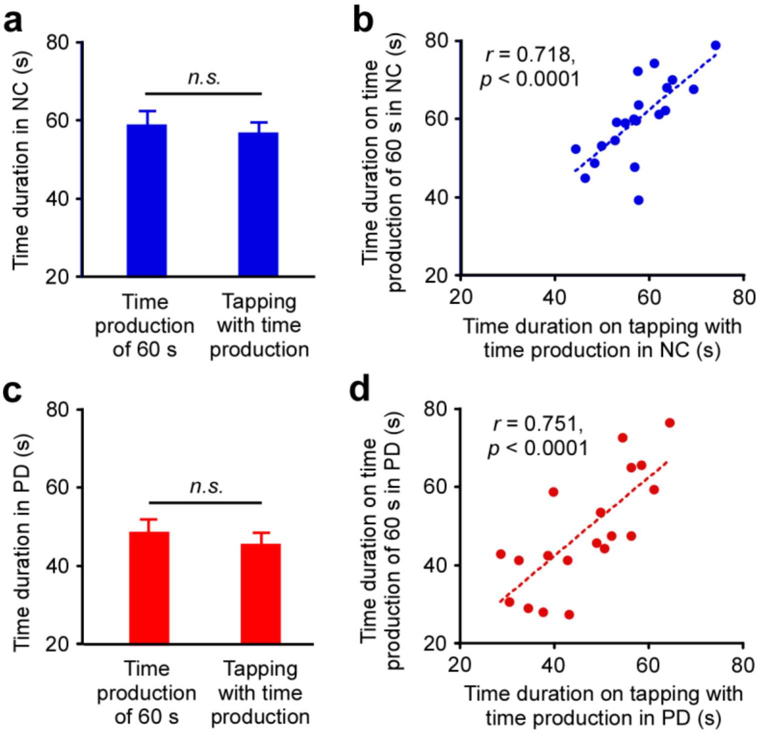

Supplementary Figure 1 | No effect of tapping action on time production.

Interval duration on time production task of 60 s was no different from those on the tapping task with time production in (**a**) normal control (NC) and (**c**) Parkinson's disease (PD). Interval durations on time production task of 60 s correlated to those on the tapping task with time production in (**b**) NC and (**d**) PD. Error bars indicate the SEM. Within individuals, estimated durations between the two tasks was nearly identical.

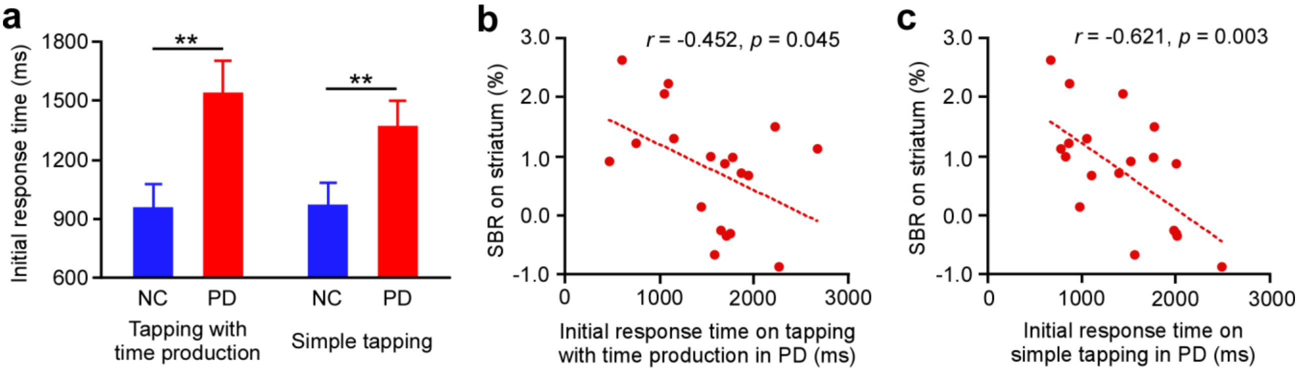

Supplementary Figure 2 | Delayed initial response in patients with PD.

(**a**) Mean initial response time in patients with Parkinson's disease (PD) was longer than normal controls (NC) on both tapping tasks. Response times in patients with PD correlated to the SBR on striatum in (**b**) the tapping task with time production (**c**) and simple tapping task. Asterisks indicate significance (**$p < 0.0001$). Error bars indicate the SEM.

Supplementary Table 1 | Correlation of time estimation with UPDRS or SBR.

	UPDRS		SBR	
	r	*p*	*r*	*p*
Time production				
0.5 s	0.307	0.202	-0.419	0.083
1 s	0.087	0.719	-0.330	0.182
2 s	0.113	0.645	-0.217	0.386
3 s	0.107	0.662	-0.255	0.307
5 s	-0.445	0.056	0.114	0.653
10 s	-0.617	0.005*	0.499	0.030*
20 s	-0.633	0.004*	0.534	0.018*
30 s	-0.713	0.001*	0.519	0.023*
60 s	-0.680	0.001*	0.561	0.012*
120 s	-0.637	0.003*	0.503	0.028*
300 s	-0.537	0.018*	0.549	0.015*
Time reproduction				
0.5 s	0.275	0.255	-0.104	0.682
1 s	0.137	0.577	-0.214	0.393
2 s	0.118	0.632	-0.327	0.186
3 s	-0.143	0.558	-0.291	0.242
5 s	-0.288	0.232	-0.217	0.386
10 s	-0.006	0.980	-0.396	0.104
20 s	-0.006	0.979	-0.113	0.656
30 s	0.197	0.419	-0.387	0.113
60 s	0.189	0.439	0.083	0.744
120 s	0.223	0.360	-0.032	0.901
300 s	0.487	0.092	-0.262	0.293

UPDRS: Unified Parkinson's Disease Rating Scale. SBR: Specific Binding Ratio on DaT imaging. Asterisks indicate significance.

Supplementary Table 2 | Correlation of time estimation with PD severity.

	UPDRS-1	UPDRS-2	UPDRS-3	UPDRS-4	Hoehn-Yahr	PD duration
Time production						
0.5 s	0.146	0.133	0.312	0.259	0.297	0.671
1 s	0.903	0.611	0.758	0.943	0.885	0.906
2 s	0.263	0.286	0.746	0.305	0.982	0.578
3 s	0.371	0.426	0.672	0.357	0.999	0.770
5 s	0.574	0.091	0.076	0.056	0.054	0.085
10 s	0.686	0.014*	0.002*	0.099	0.016*	0.069
20 s	0.938	0.021*	0.001*	0.094	0.014*	0.068
30 s	0.372	0.001*	< 0.0001*	0.072	0.018*	0.073
60 s	0.811	0.004*	0.001*	0.053	0.003*	0.155
120 s	0.428	0.005*	0.002*	0.183	0.032*	0.150
300 s	0.061	0.023*	0.018*	0.240	0.003*	0.087
Time reproduction						
0.5 s	0.117	0.160	0.286	0.942	0.743	0.890
1 s	0.750	0.597	0.606	0.556	0.874	0.573
2 s	0.675	0.634	0.578	0.856	0.684	0.536
3 s	0.484	0.554	0.569	0.810	0.685	0.228
5 s	0.156	0.095	0.349	0.672	0.917	0.208
10 s	0.760	0.771	0.968	0.621	0.308	0.226
20 s	0.285	0.756	0.869	0.872	0.919	0.159
30 s	0.621	0.537	0.246	0.644	0.166	0.936
60 s	0.487	0.451	0.420	0.841	0.367	0.719
120 s	0.940	0.499	0.248	0.879	0.096	0.881
300 s	0.068	0.083	0.130	0.140	0.453	0.091

UPDRS-1: Mentation, behavior, and mood. UPDRS-2: Activities of daily life. UPDRS-3: Motor Examination. UPDRS-4: Complications of therapy. The numbers mean *p* value of Pearson's correlation coefficient. Asterisks indicate significance.

Supplementary Video 1 | A representative trial on tapping task with time production in a patient with PD.

Although a patient was asked to make the 60 s duration in cycles of 1 s tapping, the tapping speed was considerably faster. As the result, the patient terminated at approximately 40 s, rather than the target 60 s.
